# Influence of Food Environments on Dietary Habits: Insights from a Quasi-Experimental Research

**DOI:** 10.3390/foods13132013

**Published:** 2024-06-26

**Authors:** Terrence W. Thomas, Murat Cankurt

**Affiliations:** Department of Agribusiness, Applied Economics and Agriscience Education, North Carolina A&T State University, Greensboro, NC 27411, USA; mcankurt@ncat.edu

**Keywords:** dietary habits, food environment, food desert, quasi-experimental design, ordered logistic regression

## Abstract

Nutrition is a vital factor that exerts a profound and direct impact on health. Food environments significantly influence individuals’ dietary behaviors, health outcomes, and overall food security. Individuals in food deserts and food swamps do not have access to healthier food options. And in both cases, the emphasis is primarily on the physical configuration of the environment as it relates to food availability. This quasi-experimental study aims to investigate the impact of two different food environments (defined to include a social component) on food choices. A total of 246 participants were surveyed by telephone, half of whom were primed with Scenario A (a food environment deficient in healthy options and cues that support and reinforce healthier choices) and half were primed with Scenario B (an environment with an abundance of healthy food options and cues that support and reinforce healthy eating). Ordered logit regression was used for analysis. The results show significant differences in likely food consumption between the groups. Individuals in Scenario B were found to be 4.48 times more likely to consume fruits and vegetables. In addition, it was determined that being a woman increases the probability of consuming more fruits and vegetables by 0.92 times (1/0.52–1), and adherence to a healthy diet increases by 3.64 times. Age and race were not significant predictors. This study highlights the crucial role of environmental factors in shaping dietary habits and underscores the importance of the social components of the food environment in promoting the adoption of healthier dietary habits. Based on these findings, policymakers should prioritize developing strategies that go beyond providing physical access and consider social aspects of the environment in promoting healthy eating habits to improve public health and bolster the food security of communities.

## 1. Introduction

A healthy society needs healthy individuals. The relationship between nutrition and health is undeniable. Ensuring a population’s food security is crucial for overall well-being and disease prevention. Food security, as defined by the Food and Agriculture Organization (FAO), refers to the state in which all people, at all times, have physical, social, and economic access to sufficient, safe, and nutritious food that meets their dietary needs and food preferences for an active and healthy life [1]. Food deserts and food swamps are areas where there are restrictions in access to healthy foods, such as fruits and vegetables. A food desert is typically an urban area or rural area where it is difficult to access affordable and nutritious food. These areas often lack grocery stores, supermarkets, and farmers’ markets, making it challenging for residents to obtain fresh fruits, vegetables, and other healthy foods [2]. Residents in these areas rely on convenience stores or fast-food restaurants, which tend to offer primarily processed and unhealthy options [3].

Food environments, food retail, and promotional practices influence the purchase and consumption of food and may lead to adverse health impacts attributed to diet-related chronic diseases, especially in low-income communities [4,5,6]. More recent research [7] suggests that action should be taken to modify food environments to support heathier food choices, implying that food environments play a role in influencing purchase and consumption decisions. Although other reviews [8,9] indicate that the food environment–behavior relationship is not conclusive, the result of research in this vein is decidedly mixed. Ref. [10] observed that the relationship between food environments and choice behavior is a complex one, which may explain the relative lack of success in demonstrating a strong relationship between food environments and food choice behavior. Nevertheless, the idea that behavior is influenced by the immediate environment [11,12] continues to drive investigation into how food environments impact food choice behavior. 

In the literature, researchers have investigated food environments from the perspective of the influence of food deserts, food swamps, and food retail services on choice behavior and the health impacts that flow from such choices. Food deserts, based on the USDA [13] definition, are low-income and low-access communities. Low-income communities are those with a poverty rate of 20% or greater or a median family income at or below 80% of the median family income of the area, while access is defined as those instances where at least 500 persons and 33% of the census tract population live further than a mile from a grocery store or supermarket. And food swamps are areas where there is a high concentration of fast food and convenience stores, crowding out access to heathier foods [14]. The retail food environment comprises food establishments where consumers eat out or sell food that the consumer prepares at home [15]. The foregoing concepts of food environments capture the various configurations of physical environments in which food is available and made available to the consumer. Refs. [15,16] classify food environments as economic, political, and socio-cultural, which goes beyond just the physical perspective of food environments to offer a broader view. As Ref. [15] notes, including a sociological perspective in the study of food environments could enrich our understanding by expanding the interpretation of food environments beyond their physical features. One approach involves employing social practice theory [17,18] to analyze and interpret the salience of food environments and to use [19] the conceptualization of social learning to complement the potential contribution of social practice theory to our broader understanding of food environments. This paper draws on the interpretation of practice theory as [18] describes it. Ref. [19] defines social practice as a routinized form of behavior comprising several interconnected elements that include material things and their use, physical and mental activities, and background knowledge such as know-how, understanding, emotional states, and motivational knowledge, as quoted in [18]. This definition and the perspective of [18] informs our interpretation of social practice for the purpose of this paper, that is, a practice is an accepted way of doing things shaped over time by social, political, economic, and technological forces. Furthermore, the components or elements of a practice interact to define the practice, and it evolves over time as linkages among the elements are redefined. This view of social practice separates it from practice as performance, which, in this sense, is repetition of an activity with the aim of getting better at performing the activity; choir practice comes to mind. Note that practice as performance takes place within the tradition/protocol of a particular practice, which is practice as an entity [17,18]. For example, choir practice happens within the practice of worship in the Christian religion. Practice an entity, as Refs. [17,18] describe it, can also be viewed as a system where interconnected and interacting elements shape the character of the system. Given this perspective, the system of social practice is a dynamic entity, which means that food choice behavior and the impact of this behavior are emergent outcomes, and the food environment is one element of the material component of the social practice system. Because the outcomes of this “social practice system” for example, food choice behavior and its impact, are emergent phenomena, it is difficult for researchers to connect them to a single component. This means that an analytical approach that takes a system view of food environments is likely to yield more fruitful results. Refs. [17,18] discussed using “social practice conceptualized as an entity” as the unit of analysis. The system view supports their position.

Furthermore, as [20] notes, individuals live in a network that impacts their behavior. Others, such as [21,22], argue that our social networks determine our beliefs and behaviors. However, ref. [19] provides convincing rationale and empirical evidence that support the role of social structure as a major driver of norms, habits, preferences, and behavior. Ref. [19] argued that learning from examples of other people’s behavior and the associated contextual features is an important and likely dominant mechanism for behavior change. In his family and friends study, Ref. [19] showed that exposure to the examples of peers had the greatest effect on behavior change, including health behaviors and the adoption of habits and preferences.

Considering the logic of the forgoing discussion, a more robust interpretation of food environments is called for; one that views food environments from the vantage point of “social practice theory” and [19] the exposition of social structure (engagement, social learning, and exposure). Seeing food environments as “a social practice”, that is, a practice as an entity, would draw attention to the emotions, preferences, and values that are shaped from the interactions among the components of the “social practice” of purchasing, preparing, and consuming food, the social interaction around food, the resultant social learning, and the myriad of cues in the environment that reinforce or discourage healthy eating habits, for example, the comments of friends and family, observations of their behavior, network effects, and the resultant social learning.

So, addressing the complex issue of food environments requires giving attention to more than physical access. Cues in the environment, such as those described in Scenarios A and B, need consideration for formulating strategies to improve dietary choices and eating habits.

Guilford County, North Carolina, has large pockets of low-income communities classified as food deserts. According to the North Carolina Department Health and Human Services, 8% of North Carolina residents (715,223 people) have limited access to healthy foods [23]. The relatively high incidence of chronic diseases among low-income food desert residents is often attributed to an unhealthy lifestyle associated with a lack of physical activity and poor eating habits. This study includes the results of the analysis of data obtained from a random sample of Guilford County residents in North Carolina.

The aim of this study was to investigate the direct impact of two different food environments, as defined in Scenarios A and B, on individuals’ dietary behaviors using a quasi-experimental research design. We also had some subobjectives under the main objective:

- To determine the likelihood of individuals adopting healthy eating behaviors in two different food environments, as defined in Scenarios A and B.
- To determine the factors and their weights influencing the likelihood of consuming more fruits and vegetables.

What makes this study important is that it extends the definition of food environments to include socio-cultural factors, which deepens the salience of the food environment relative to the lived experience of residents as depicted in the scenarios. Previous studies have emphasized the physical aspects of environments, defined as food retail environments, food deserts, or food swamps. By employing a quasi-experimental research design and considering two contrasting scenarios, this research not only expands our understanding of the direct influence of food environments but also emphasizes the significance of such influences on dietary habits. The findings, derived from a comprehensive analysis of participants in Guilford County, North Carolina, using an ordered logit regression model, contribute valuable insights to the pivotal role that environmental factors play in shaping food choices. This study carries implications for public health policies and interventions, offering a nuanced perspective on how food environments that more broadly reflect the lived experience of residents can be instrumental in fostering and sustaining healthier dietary behaviors among individuals.

## 2. Materials and Methods

### 2.1. Methods Used in Obtaining the Data

This study employed a quasi-experimental approach, as described below. The main material of this study was original survey data obtained from a random sample of individuals living in Guilford County, North Carolina. In obtaining the data, a sample size of 280 was determined according to a 95% confidence interval and a 6% margin error, using the proportional sample size calculation formula by Nelson [24]. The sampling firm Dynata (4 Research Drive, Suite 300, Shelton, CT, USA) used our sampling protocol to draw a random sample from the defined population. Two experienced enumerators collected the data via telephone calls. One enumerator administered Scenario A and the second enumerator administered Scenario B. The enumerators selected telephone numbers from the list Dynata provided.

We designed the survey questionnaire using the Qualtrics online platform to conduct the interviews.

Despite its apparent simplicity, this method required more than ten attempts to complete a single survey. In total, nearly 3000 attempts were made. The responses obtained from individuals were recorded online in the Qualtrics database. The data downloaded from the database were reviewed and cleaned—removing inconsistent surveys and adjusting for missing data to ensure that the analyses were unbiased and that consistent data were prepared for the analysis [25].

Removing inconsistent surveys from a dataset is a crucial step to enhance the quality of survey data and ensure the reliability of results. Inconsistencies can arise from contradictions, denials, or illogical responses. Eliminating such outliers contributes to establishing a more solid foundation for analysis. A study conducted at the University of North Carolina suggested that excluding inconsistent or misleading data from analysis can enhance result reliability [26]. Removing inconsistent data to maintain data integrity and improve the accuracy of analysis is a common practice [27].

With the cleaning process of the data, the margin of error of the sample in representing the population increased only slightly, not significantly. After weeding out the questionnaires with outliers and missing data, the sample amount decreased to 246. This sample size represents a population with a 95% confidence interval and a margin of error of 6%. Of these participants, half (123) were administered Scenario A, and half (123) were administered Scenario B [28].

Scenario A

Narrative 1: In several communities, the food environment is comprised of convenient stores that sell only packaged foods high in fat, sugar, and salt. They do not sell fresh fruits and vegetables. There are no roadside stands that offer fresh fruits and vegetables, and no full-service supermarkets that sell fresh fruits and vegetables. Churches and other organizations serve meals that do not include fresh fruits and vegetables, and advertisements on television promote fast foods. These ads do not promote eating more fruits and vegetables. Community members say that they and their friends are doing well without eating more fruits and vegetables, and it is too expensive to buy fruits and vegetables. They also feel that they are in better physical shape than many of the people encouraging them to eat more fruits and vegetables. Community members say that from what they see and hear around them in the community, it is not clear to them that eating more fruits and vegetables brings any health benefits. And they do not know any friends that have died or gotten sick because they did not eat more fruits and vegetables.

Scenario B

Narrative 2: In several communities, the convenience stores have recently stocked increased amounts of fresh fruits and vegetables, and the number of mobile food vendors and roadside stands selling fresh fruits and vegetables in the community have doubled. Community leaders, pastors of churches, community health specialists, and doctors have pleaded with the people to eat more fresh fruits and vegetables. Local organizations that provide hot meals, such as churches and food banks, have increased the amount of fresh fruits and vegetables served with each meal. All the experts and community leaders and friends insist that eating more fresh fruits and vegetables leads to better health, better results in medical check-ups, and people remaining very active with fewer complaints as they get older. Just like medicine, fruits and vegetables work over an extended period to bring relief and benefits, but fruits and vegetables cost less and taste better. In all these communities, the reports show that residents spent 50 per cent less money on visits to doctors and take far fewer prescription drugs for controlling blood pressure, diabetes, and cholesterol. Plus, several community members have developed thriving businesses to supply the increased demand for vegetables and fruits.

The respondents were asked to answer the questions outlined below following the administration of Scenario A and Scenario B. To make a robust comparison, both groups of participants were asked the same questions. In other words, an experimental setup was created in which only the food environment they were in changed, but the questions were the same. This was to determine whether the environment influenced dietary habits.

Basic questions (considering the stories told above):

Q1. Would you eat more fruits and vegetables if you lived in such a community?
  (H0: The amount of fruit and vegetable eating does not change according to different food environments.)
Q2. To what extent do you believe eating more fruits and vegetables would lead to improvement in your health?
  (H0: The belief that eating more fruits and vegetables would improve health does not change according to different food environments.)
Q3. To what extent would you be committed to eating more fruits and vegetables if you were living in the community described in the story.
  (H0: The determination to continue eating more fruits and vegetables does not change according to different food environments.)
Q4. What percentage of the residents in these communities do you think would be eating more fruits and vegetables?
  (H0: The percentage of people in the community who consume more fruits and vegetables does not change according to different food environments.)

### 2.2. Methods Used in Data Analysis

The quasi-experimental research design, which was created in line with the aim of the study, focused on whether two different food environments create differences in the dietary habits of individuals [28,29]. All data analyses were conducted in SPSS 29.0 (IBM Corp., Armonk, NY, USA). to see whether there were statistically significant differences between the respondents responding to Scenario A and Scenario B.Before deciding on the statistical tests, it was determined whether our data were parametric or not. For this purpose, a normality test was performed, and it was determined that all variables except age were not normally distributed (Kolmogorov–Smirnov: *p* < 0.001). Therefore, it was decided that the tests to be used should be non-parametric tests.

Since there was a statistically significant difference between the groups, a regression analysis was performed to determine the factors affecting the likelihood of consuming more fruits and vegetables (MVFCProb). Since the data structure of the dependent variable was ordinal, the ordered logit regression model, one of the ordinal dependent variable models, was used.

### 2.3. Ordered Logit Regression Model

The ordered logit model is a statistical regression model used to analyze ordinal dependent variables that have a meaningful order and more than two categories [30]. Ordinal variables have categories with a natural order but with unknown or unequal intervals between them. The ordered logit model is an extension of the binary logit model and is particularly useful when the outcome variable has more than two ordered categories [31]. Models in the analyses were estimated by the ordered logit regression (OLR). OLR is a statistical technique that can sometimes be used with an ordered (from low to high) dependent variable [32].

The ordered logit model assumes a linear relationship between the explanatory variables and the cumulative odds of falling into a particular category [33]. Let us consider a dependent variable *Y* with *J* ordered categories. The cumulative odds of falling into category *j* are given by:

$$logit\left(P\left(Y\leq j\right)\right)=\alpha_{j}+\beta_{1}X_{1}+\beta_{2}X_{2}+{\ldots +\beta }_{k}X_{k}$$

where:

- *P*(*Y* ≤ *j*) is the cumulative probability of *Y* being less than or equal to *j*.
- *α_j_* is the intercept for category *j*.
- *β*_1_, *β*_2_, …, *β_k_* are the coefficients for the explanatory variables 1, 2, …, *X*_1_, *X*_2_, …, *X_k_*.

The model parameters are estimated using maximum likelihood estimation (MLE). The likelihood function is maximized to find the parameter values that make the observed data most probable under the assumed model [34,35].

This model is known as the proportional odds model because the odds ratio of the event is independent of the category *j*. The odds ratio is assumed to be constant for all categories. It simultaneously estimates multiple equations. The number of equations it estimates is the number of categories in the dependent variable minus one. OLR provides only one set of coefficients for each independent variable. Therefore, there is an assumption of parallel regression, or, as it is called, the proportional odds assumption. Ordinal logistic regression assumes that the coefficients that describe the relationship between the lowest versus all higher categories of the response variable are the same as those that describe the relationship between the next lowest category and all higher categories. That is, the coefficients for the variables in the equations would not vary significantly if they were estimated separately. The intercepts would be different, but the slopes would be essentially the same in only one model [36]. If this was not the case, we would need different models to describe the relationship between each pair of outcome groups. We needed to test the proportional odds assumption. The null hypothesis of this chi-square test is that there is no difference in the coefficients between the models.

The ordered logit model is widely used in various fields, including economics, social sciences, and public health, to analyze and understand the factors influencing ordinal outcomes. Researchers often rely on its flexibility and interpretability to gain insights into the relationships between variables and ordered categorical responses.

The dependent variable used in this study was the likelihood of eating more fruits and vegetables. The estimated values were used to estimate a response value [37].

## 3. Results and Discussion

The basis of the research design was to determine whether two different food environments caused behavioral differences in likely food consumption among individuals in the community. If a behavioral change was found to occur, it was also investigated which individuals influenced this behavioral change and to what extent.

For a better understanding of the variables used in the analyses, their detailed information is given in Table 1.

For the purpose of our research, we first tested whether there was a statistically significant difference between the different food environments, i.e., Scenario A and Scenario B, in terms of other variables. 

The Z-value in the Mann–Whitney test served as a test statistic, quantifying the disparity between two independent samples. It was computed by dividing the rank sum difference of the two samples by the square root of half the product of their respective sample sizes.

As seen in Table 2, there was a big difference between the less healthy food environment (Scenario 1) and the healthier food environment (Scenario 2) in terms of the variable MVFCProb, which shows the probability of consuming more fruits and vegetables. While the mean of the first group was 3.93, this value increased to 6.19 in the second group. If we look at the Z score produced by the Mann–Whitney *U* test (−9.56), the difference between the groups is not random, but statistically significant. This means that individuals are extremely likely (7) to consume fruits and vegetables in a food oasis compared to in a food desert (4).

For the variable BtrHProb, which represents believing that consuming more fruits and vegetables would improve health, the difference was statistically significant, although the means were close to each other in both groups. In other words, there was a difference between the two groups. In Scenario B, people are more likely to believe that eating more fruits and vegetables is beneficial for their health compared to Scenario A. However, it is noteworthy that the mean of both groups was above 6, and the median was 7. This means that both groups of respondents thought that fruits and vegetables are good for their health.

When the answers to the question “If you lived in the kind of society described in the scenario, to what extent would you be determined to eat more fruits and vegetables (MVFLoyal)*?*” were analyzed, it was observed that there was a difference between the groups, and the difference was statistically significant. The individuals responding to Scenario B (food oasis) had a high level of commitment (7) to consume more fruits and vegetables. In the case of individuals responding to Scenario A (food desert), the respondents stated that they would remain moderately loyal (4).

According to the scenarios given, when the answers to the question “What percentage of the residents in these communities do you think would be eating more fruits and vegetables?” (ComCPercMVF) were analyzed, it was seen that the difference between the groups was significant. The respondents given Scenario A (food desert) were likely to think that 36% of their communities would be eating more fruits and vegetables, compared to 50% in Scenario B (food oasis).

When the variables of age and gender are analyzed, it is seen that there was no statistically significant difference between the groups. In other words, the individuals responding to Scenario A did not differ in the values of the age and gender variables from those responding to Scenario B. However, there was a difference between the groups in the variable of race (Race3). In a study such as this, where sampling is carried out well and data are collected without errors, it is not expected that the age and gender of individuals in different food environments will change.

According to the results, there are statistically significant differences between the two food environments in the MVFCProb, BtrHProb, MVFLoyal, ComCPercMVF, and Race3 variables. On the other hand, there was no significant difference between the groups in the age and gender variables. Except for gender and age, the statistical difference between the groups was highly significant (*p* < 0.01).

In summary, there are differences in the likelihood of behavior and opinions based on differences in food environments. Individuals in healthier and supportive food environments will likely consume more fruits and vegetables, they believe that this consumption is likely to be more beneficial to their health, and they are more likely to commit to consuming more fruits and vegetables than individuals in less healthy and supportive food environments.

The main question of the research was whether different food environments influence individuals’ dietary behavior. It was hypothesized that individuals were more likely to consume more fruits and vegetables in a healthier food environment rich in positive and supportive cues, as described in Scenario B, than those individuals exposed to the opposite environment described in Scenario A.

Therefore, MVFCProb was assigned as a dependent variable to identify the factors influencing the likelihood of consuming more fruits and vegetables. In addition to the gender, age, and race variables, FoodEnvD (representing the different food environments), MVFLoyal (representing the commitment to consuming more fruits and vegetables), and BtrHProb (representing the belief that consuming more fruits and vegetables will lead to better health outcomes) were also included in the model as independent variables in the ordered logit model.

When the goodness of fit values of the model (Table 3) are analyzed, it is seen that the model is significant and interpretable. According to the ordered logit model results, the age, Race3 and BtrHProb variables were not statistically significant as in [39]. While many studies have shown a significant association between age and demand for healthy food [40,41,42], other studies show that younger generations, especially millennials and Generation Z, are increasingly interested in health-conscious diets and organic and healthy food options [43,44]. In our study, we did not find a significant difference in the likelihood of more demand for healthy food. However, FoodEnvD, MVFLoyal, and GENDER were statistically significant variables.

Firstly, the FoodEnvD variable, which represents the different food environments, the focus of this research, was the most effective factor in the likelihood of consuming more fruits and vegetables. In other words, as environmental conditions change toward heathier and more supportive environments, the likelihood of individuals consuming more healthy fruits and vegetables increases. According to the model results, an individual in Scenario B (health food oasis) is approximately 4.5 times more likely to consume more fruits and vegetables than an individual in Scenario A (food desert); see also Ref. [45]. Research has shown that the consumer dietary environment, including factors such as food availability, price, quality, and labeling, differs significantly between food deserts and food oases. According to demand theory, price is one of the most important factors influencing purchase. However, when comparing different environmental conditions, the prices of healthy products are not significantly different from other options, indicating that affordability is less of a barrier than availability and the social context of the environment (Pentland) in these regions, further underscoring the importance of the food environment [45], especially when the social component is considered. As Ref. [20] observed, exposure (advertisers take advantage of this fact) and social learning exert powerful influences on behavior. People are socially connected and learn from each other via these connections. In the view of social practice theory, such learning is integrated into the “social practice” by way of the knowledge and know-how components we would call “diet-related behaviors”, so that this becomes a feature of the collective rather than the individual [17,18].

Then follows the variable MVFLoyal, which shows that if the level of commitment to consume more fruits and vegetables increases by one unit, the probability of consuming more fruits and vegetables increases by about 3.5 times. This is an expected result that is not very surprising, especially if this is considered an element of the knowledge and know-how components of the social practice system.

As in many studies, gender was found to be an influential variable on the likelihood of individuals consuming more fruits and vegetables [46,47]. Women are 48% more likely to eat fruits and vegetables than men. Compared to men, women are more likely to take care of their health, as confirmed by many studies [38,48]. The tendency of women to engage at higher levels in taking positive action to improve their health status is confirmed from our observation that women attend hands-on interactive nutrition education workshops we conducted in far greater numbers than men. And according to a previous study [12], women have more positive food-related values than men.

Nevertheless, factors like age, race, and conviction in the health advantages of consuming fruits and vegetables (BtrHProb) did not exhibit a statistically notable influence on the probability of augmenting fruit and vegetable intake. Investigating the insignificance of the variable BtrHProb reveals that all respondents strongly believe in the health benefits of consuming more fruits and vegetables. This insignificance is because the belief levels of the participants are clustered at the highest level of the 7-point scale and there is no variability. Therefore, the variable is not significant in terms of the statistical analysis. On the other hand, according to the Health Belief Model (HBM), individuals with a strong belief that their health will improve if they consume more fruits and vegetables are more likely to engage in these healthy behaviors. This belief may motivate individuals to make healthy choices, thereby helping them to make positive changes in their health behaviors and improve their overall health [49].

The findings underscore the significant role of food environmental factors, especially the social elements of food surroundings, in shaping individuals’ dietary choices. The findings highlight the potential for healthier eating habits to develop in environments supportive of nutritious food access and dietary habits [36].

## 4. Conclusions

The findings of this quasi-experimental research provide valuable insights into the influence of different food environments on individuals’ dietary behaviors and food consumption. This study, conducted in Guilford County, North Carolina, aimed to investigate whether residing in a food environment characterized by limited access and depleted of cues supportive of healthy dietary habits (food desert) influences the likelihood of consuming more fruits and vegetables compared to living in a food environment with adequate access to health options and rich in supportive social cues (food oasis). The results, derived from a sample of 246 participants, indicate notable differences in the likely food consumption behaviors between the two groups. Individuals primed with the healthy food environment scenario (Scenario B) reported a greater likelihood of increased consumption of fruits and vegetables compared to those primed with the food desert scenario (Scenario A).

The statistical analysis revealed significant differences between the two food environments for key variables. Individuals primed with Scenario B demonstrated a higher probability of consuming more fruits and vegetables (MVFCProb), a stronger belief that such consumption would improve their health (BtrHProb), and a higher commitment to this dietary behavior (MVFLoyal) compared to individuals in a food desert.

Furthermore, the ordered logit regression model was used to examine the effects of various factors on the likelihood of individuals consuming more fruits and vegetables. The key findings of the ordered logit model are on environmental impact, commitment, and gender differences.

The different food environments (FoodEnvD) were the most influential factor, with individuals in a food oasis being 4.5 times more likely to consume fruits and vegetables compared to those in a food desert. Commitment to eating more fruits and vegetables (MVFLoyal) significantly increased this likelihood, with a one-unit rise in commitment leading to a 3.5-fold increase. Gender differences were also notable, as males were about half as likely as females to consume more fruits and vegetables.

These findings underscore the crucial role of the food environment, broadly defined to encompass a social element, as depicted in the scenarios, in shaping individuals’ dietary habits. Policymakers, public health officials, and community leaders should consider interventions that go beyond improving physical access and consider the social elements of the environment as they devise interventions and policy aimed at promoting healthier food choices and overall dietary behavior.

In this context, it is clear from the findings that individuals will consume more healthy products and, therefore, be healthier when their food environment is transformed from a food desert, as represented in Scenario A, to the environment represented in Scenario B (a food oasis). This means less health expenditure and more skilled labor. Spending in these areas will reduce the costs to the government in the medium and long term. Future research should examine the food environment from the perspective of social practice theory, that is, investigating “dietary behavior” as a social practice and as the unit of analysis. This perspective would enrich our view of food environments to include the diverse social components of the system from network effects to social learning and exposure. Components of food environments include not only physical access (the availability of super markets, farmers markets and grocery stores) and economic access (food prices and household income), food quality (availability of fresh, unprocessed foods), but most importantly, the socio-cultural factors (customs, networks, social learning, and exposure and social engagement on food choices), and political–regulatory frameworks (laws and policies affecting access to healthy food) [50]. These components directly influence individuals’ capacity to make healthy food choices. Therefore, strategies that build on these will provide more effective solutions. This approach would also bring attention to the activities focusing on housewives, who are often the determinants of household food consumption, and their role in social networks, social learning, and exposure.

In conclusion, addressing disparities in food access and promoting a supportive environment that includes socio-cultural and political factors of healthy food choices can play a pivotal role in fostering improved dietary behaviors and, ultimately, enhancing public health outcomes.

## Figures and Tables

**Table 1:** Descriptions of the variables.

Variable Name	Description of the Variable	Data Type
FoodEnvD	1 is dummy of Scenario B. Zero represents the environment of Scenario A.	0–1
MVFCProb	How likely is it that you would eat more fruits and vegetables if you were a resident in such a community described in the scenario?	7-point ordinal scale *
BtrHProb	To what extent do you believe eating more fruits and vegetables would lead to improvements your health if you were a resident in such a community described in the scenario?	7-point ordinal scale
MVFLoyal	To what extent would you be committed to eating more fruits and vegetables if you were a resident in such a community described in the scenario?	7-point ordinal scale
ComCPercMVF	What percentage of the residents in these communities do you think would be eating more fruits and vegetables?	%
Age	Age of the respondent in years.	Year
Gender	1 is dummy of male and zero represents female.	0–1
Race3	Indicates the race of the participant. 1 is White, 2 is African American, 3 is Other (less than 5% of participants were considered in this group).	3 Groups

* On a 7-point ordinal scale, one (1) represents an extremely low likelihood, and seven (7) an extremely high likelihood. Since the research data were ordinal and not normally distributed, we used non-parametric statistical tests for group comparisons. Since two independent groups were compared, the Mann–Whitney *U* test was used [38].

**Table 2:** Descriptive statistics and group comparison test.

Variables	Scenario Groups	Mean	Median	Min	Max	Std. Dev.	Z	Sign
MVFCProb	A	3.93	4.00	1.00	7.00	1.74	−9.56	***
	B	6.19	7.00	1.00	7.00	1.25		
BtrHProb	A	6.20	7.00	1.00	7.00	1.08	−5.60	***
	B	6.83	7.00	4.00	7.00	0.52		
MVFLoyal	A	4.31	4.00	1.00	7.00	1.67	−8.17	***
	B	6.24	7.00	1.00	7.00	1.22		
ComCPercMVF	A	35.70	30.00	5.00	85.00	23.02	−4.30	***
	B	50.01	50.00	1.00	100.00	26.36		
Age	A	49.70	48.00	23.00	88.00	14.60	−0.42	
	B	50.79	51.00	18.00	88.00	16.75		
Gender								
Female (0)	A	68						
	B	58					−1.27	
Male (1)	A	55						
	B	65						
Race3								
White (1)	A	65						
	B	84						
African American (2)	A	53					−2.28	**
	B	33						
Others (3)	A	5						
	B	6						

*** *p* < 0.001; ** *p* < 0.01 are significance level.

**Table 3:** Ordered logit model on likelihood of consuming more fruits and vegetables.

Variables MVFCProb (Dependent Var)	Coefficient	Odds Ratio	Std. Error	z	*p*-Value	
FoodEnvD	1.501	4.486	0.311	23.351	<0.001	***
MVFLoyal	1.291	3.636	0.118	119.107	<0.001	***
GENDER	−0.654	0.520	0.258	6.408	0.011	***
AGE	−0.015	0.985	0.185	0.007	0.934	
RACE3	0.219	1.245	0.229	0.912	0.340	
BtrHProb	0.078	1.081	0.147	0.28	0.597	
(MVFCProb = 1.00)	2.371		1.089	4.736	0.03	**
(MVFCProb = 2.00)	3.967		1.078	13.552	<0.001	***
(MVFCProb = 3.00)	5.303		1.094	23.478	<0.001	***
(MVFCProb = 4.00)	6.552		1.125	33.904	<0.001	***
(MVFCProb = 5.00)	8.476		1.197	50.128	<0.001	***
(MVFCProb = 6.00)	9.798		1.237	62.698	<0.001	***

*** *p* < 0.001; ** *p* < 0.01 are significance level. Parallel line test: Chi-square (30) = 40.241. Likelihood ratio test: Chi-square (6) = 271.088 [<0.001]. Pseudo R-Square = 0.69.

## Data Availability

The original contributions presented in the study are included in the article, further inquiries can be directed to the corresponding author.
